# Integration of the cancer-related inflammatory response as a stratifying biomarker of survival in hepatocellular carcinoma treated with sorafenib

**DOI:** 10.18632/oncotarget.15322

**Published:** 2017-02-14

**Authors:** Jessica A. Howell, David J. Pinato, Ramya Ramaswami, Tadaaki Arizumi, Carlotta Ferrari, Antonello Gibbin, Michela E. Burlone, Giulia Guaschino, Pierluigi Toniutto, James Black, Laura Sellers, Masatoshi Kudo, Mario Pirisi, Rohini Sharma

**Affiliations:** ^1^ Department of Surgery and Cancer, Imperial College London, Hammersmith Hospital, London, UK; ^2^ Centre for Population Health, Burnet Institute, Melbourne, Australia; ^3^ Department of Medicine, University of Melbourne, Melbourne, Australia; ^4^ Department of Gastroenterology and Hepatology, Kinki University School of Medicine, Osaka-Sayama, Osaka, Japan; ^5^ Department of Translational Medicine, Università degli Studi del Piemonte Orientale “A. Avogadro”, Novara, Italy; ^6^ Department of Experimental and Clinical Medicine, University of Udine, Udine, Italy

**Keywords:** liver cancer, VEGF inhibitor, CLIP score, BCLC, inflammation

## Abstract

**Background and Aims:**

Response to sorafenib is highly variable in hepatocellular carcinoma (HCC). Baseline inflammatory parameters and treatment toxicities may improve survival prediction in patients on sorafenib therapy.

**Results:**

442 patients with advanced stage HCC on sorafenib were recruited (follow-up 5096 person-months at risk). 88% had BCLC stage B or greater HCC and 72.3% had Child-Pugh A cirrhosis. On Cox multivariate regression, previously-treated HCC (HR 0.579, 95% CI 0.385-0.872, p=0.009), Cancer of Liver Italian Program (CLIP) score (HR 1.723, 95% CI 1.462-2.047, p<0.0001), baseline red cell distribution width (RDW; HR 1.234, 95% CI 1.115-1.290, p<0.0001) and neutrophil to lymphocyte ratio (NLR; HR 1.218, 95% CI 1.108-1.322, p<0.0001) were significant independent risks for shorter survival, whilst sorafenib-related diarrhoea was associated with prolonged survival (HR 0.533, 95% CI 0.373-0.763, p=0.001). The combination of RD-CLIP score (CLIP score multiplied by RDW) ≥ 70 and no treatment-related diarrhoea had good utility for predicting 3-month survival (AUC of 0.808 (95% CI 0.734-0.882), positive predictive value of 86.4% and negative predictive value of 83.3%), compared with CLIP (AUC=0.642) or BCLC score alone (AUC=0.579). RD-CLIP score ≥ 35 and no treatment-related diarrhoea had an AUC of 0.787 for predicting 12-month survival.

**Methods:**

Patients with HCC were consecutively recruited from three tertiary centres (Japan, Italy and UK) and clinical data were prospectively collected. The primary study endpoint was overall survival (OS) after commencing sorafenib.

**Conclusion:**

The novel prognostic index of CLIP score combined with inflammatory marker RDW and treatment-related diarrhoea has good accuracy for predicting overall, 3 month and 12 month survival in patients on sorafenib.

## INTRODUCTION

Hepatocellular carcinoma (HCC) is the sixth most common malignancy worldwide and the third most common cause of cancer-related death) [1]. Despite major advances in the diagnosis and therapy of HCC, incidence of HCC is increasing worldwide and mortality remains high despite the global trend of falling cancer death rates over the last decade [1, 2].

Sorafenib, an oral multi-targeted inhibitor of Raf, Platelet-derived (PDGF) and Vascular Endothelial Growth Factor (VEGF) receptors, has proven efficacy for advanced HCC, with a reported median 3-month improvement in patient survival and delayed radiological disease progression by an average of 2 months [3, 4]. However, in the clinical setting overall response rates to sorafenib are highly heterogeneous [3], due to the fact that mortality from HCC is not solely influenced by tumour stage, but also by underlying liver function impairment and patient performance status.

There are currently no validated stratifying biomarkers to predict sorafenib treatment efficacy. Whilst molecular traits including intra-tumour Erk phosphorylation [5] or focal gains in VEGF-A [6] have been postulated as biomarkers of enhanced sorafenib sensitivity, these have not translated into validated, accessible prognostic tests. Given drug-related toxicities and the cost of sorafenib therapy [3, 4, 7], there is an unmet need for inexpensive, accessible clinical biomarkers to help identify patients who are most likely to benefit from sorafenib.

Inflammation is a well-established driver of liver fibrosis and carcinogenesis and inflammatory parameters predict survival in both cirrhosis [8, 9] and HCC [10–14]. Gene expression studies have shown a pro-inflammatory signature in the tumour microenvironment predicts shorter time to recurrence and worse OS in patients receiving radical treatment for HCC [10]. Several groups have previously shown that inflammatory parameters, including the Inflammation-based Index (IBI) and the neutrophil to lymphocyte ratio (NLR), are associated with worse survival in HCC [11–14]. More recently, we demonstrated that stage-dependent deterioration in red cell distribution width (RDW), a marker of anistocytosis, is associated with overall survival in HCC, with the strongest prognostic role of RDW demonstrated in patients with advanced HCC [15]. Moreover, inflammatory parameters such as RDW are inexpensive and accessible biomarkers for survival prediction in HCC.

The aim of this study was to use a multivariable regression model to evaluate the prognostic value of common clinical and biochemical parameters including RDW and systemic inflammatory biomarkers in a large, prospectively recruited mixed-race cohort of HCC patients receiving sorafenib. Based on our survival analysis, we derived a composite Cancer Liver Italian Program (CLIP) and inflammation-based algorithm to optimise the prediction of survival benefit from sorafenib therapy in HCC.

## RESULTS

Four hundred and forty two patients with HCC were recruited, with a median follow-up time of 7.1 months (IQR 3.4-16.1 months) and overall follow-up time of 5096 person-months at risk. The mean age was 70 +/−10 years and 78% were male. The majority of patients in the cohort had CTP class A (73%) or B (27%) disease, with a median CTP score of 6 (IQR 5-7). With respect to tumour stage, the majority of patients had a BCLC score of B or greater (88%), 29% had portal vein thrombosis (PVT) and 24% had extra-hepatic metastases. Twenty-five percent had received loco-regional treatment prior to sorafenib. A summary of the distribution of clinical variables is outlined in Table 1.

**Table 1:** Distribution of clinical variables within the cohort (n=442)

Clinical Variable	
Number of deaths (N, %) (n=442)	311 (70.4%)
Gender N (%)(n=442)	
Male	346 (78.3%)
Female	96 (21.7%)
Mean Age (years) +/− sd (n=442)	69.92 +/− 10.06 years
Median (IQR) ALT (μmol/L) (n=438)	41 (26-70)
Median (IQR) total bilirubin (μmol/L) (n=442)	16.00 (10.30-23.94)
Median (IQR) albumin (g/L) (n=441)	36 (31-39)
Median (IQR) CTP score (n=441)	6 (5-7)
Median (IQR) neutrophils (x10^9^/ L) (n=438)	3.1 (2.3-4.4)
Median (IQR) lymphocytes (x10^9^/ L) (n=438)	1.30 (0.99-1.73)
Median (IQR) platelets (x10^9^/ L) (n=440)	145.5 (97.0-210.5)
Median (IQR) NLR (n=438)	2.52 (1.68-3.40)
Median (IQR) red cell distribution width (n=425)	14.2 (13.1-16.0)
Viral hepatitis N (%) (n=213)	98 (46.0%)
Alcohol Liver Disease N (%) (n=210)	97 (46.2%)
Tumour morphology N (%) (n=438)	
<50% uninodular	62 (14.2%)
<50% multinodular	257 (58.7%)
>50% multinodular	119 (27.2%)
Median (IQR) AFP (ng/mL) (n=420)	128 (9-1616)
Portal vein thrombosis N (%) (n=442)	128 (29.0%)
Median (IQR) Tumour size (cm) (n=427)	4.2cm (2.1-7.3)
Number of nodules N (%) (n=390)	
1-3 nodules	174 (44.5%)
4-6 nodules	100 (25.6%)
7-10 nodules	102 (26.2%)
Multinodular	8 (2.1%)
Diffuse	6 (1.5%)
Metastases N (%) (n=442)	108 (24.4%)
BCLC score N (%) (n=428)	
A1	12 (2.8%)
A2	2 (0.5%)
A3	3 (0.7%)
A4	34 (7.9%)
B	148 (34.6%)
C	225 (52.6%)
D	4 (0.9%)
Median (IQR) CLIP score (n=276)	2 (1-3)
Treatment-naïve versus previously treated HCC (n=204)	
Treatment-naïve HCC	103 (50.5%)
Previously treated HCC	101 (49.5%)
Median (IQR) Sorafenib duration (months) (n=440)	3.97 (1.63-10.30)
Adverse events from sorafenib*:	
Diarrhoea (n=261)	156 (59.78%)
Hypertension (n=168)	77 (45.8%)
Hand-foot syndrome (n=287)	148 (51.7%)
Mucositis (n=118)	12 (10.2%)

^*^ Adverse events from sorafenib developing during any cycle of treatment

IQR, interquartile range; ALT, alanine aminotransferase; CTP, Child Turcotte Pugh score; NLR, neutrophil to lymphocyte ratio; BCLC, Barcelona Clinic Liver Cancer score; CLIP, Cancer of the Liver Italian Program.

### Clinical variables associated with overall survival after sorafenib therapy

Median duration of sorafenib therapy was 3.97 months (IQR 1.6-10.3 months), with the majority of patients ceasing therapy due to progressive disease (178 of 438 patients in whom follow up data were available, 40.6%) or unacceptable toxicity (111 of 438, 25.3%). Overall, 311 (70.4%) patients died during follow up, with a median survival time of 9.6 months (IQR 4.3-21.3 months) after commencing sorafenib. Three-month survival was 78.5% (346/441) and twelve-month survival was 33% (147/441). Overall response to sorafenib (either complete, partial disease response or stabilisation of disease without radiological progression) occurred in 221 (50.2%) patients, whereas 221 (49.8%) developed progressive HCC on imaging during follow up. The vast majority (363/ 442, 82.1%) of patients experienced at least one side effect of grade 1 or higher severity.

Univariate predictors of OS included age (p=0.013), baseline CTP score (p<0.0001), NLR (p<0.0001), RDW (p<0.0001), presence of portal venous thrombosis (PVT, p<0.0001), presence of metastases (p=0.002), CLIP score (p=0.001) and BCLC score (p=0.009). Patients who were treatment naïve had longer survival compared to patients who had previously undergone therapy (p=0.006). Moreover, development of side effects on sorafenib (of any severity and at any treatment stage), including hypertension p<0.0001, hand-foot syndrome p<0.0001 and diarrhoea p<0.0001 were univariate predictors of survival (Supplementary Table 1). However, on Cox proportional hazards analysis (n=175 patients who had complete data for all parameters), treatment-naïve compared with previously treated HCC (HR 0.579, 95% CI 0.385-0.872, p=0.009), CLIP score (HR 1.723, 95% CI 1.462-2.047, p<0.0001), RDW (HR 1.234, 95% CI 1.115-1.290, p<0.0001) and NLR (HR 1.218, 95% CI 1.108-1.322, p<0.0001) were all significant independent risks for shorter survival in patients receiving sorafenib. In contrast, sorafenib-related diarrhoea was associated with prolonged survival (HR 0.533, 95% CI 0.373-0.763, p=0.001; Table 2).

**Table 2:** Summary of clinical variables independently associated with survival in patients on sorafenib therapy: results of Cox proportional hazards multivariate analysis (n=175)

Clinical Variable	HR	95% CI	P-value
Treatment-naïve HCC	0.579	0.385-0.872	0.009
NLR	1.252	1.139-1.377	<0.0001
RDW	1.136	1.056-1.223	0.001
CLIP score	1.383	1.195-1.600	<0.0001
Diarrhoea on sorafenib	0.596	0.417-0.852	0.005

NLR, neutrophil to lymphocyte ratio; RDW, red cell distribution width; AFP, alpha-fetoprotein; CLIP score, Cancer of the Liver Italian Program score. Primary tumour refers to primary diagnosis of tumour, compared with recurrent tumour after previous therapy.

### RD-CLIP score and diarrhoea predicts three-month survival in patients receiving sorafenib

We further evaluated clinical variables associated with survival endpoints at 3 and 12 months. There was a significant association between 3-month survival and lower CLIP score (OR 0.522, 95% CI 0.382-0.714, p<0.0001), lower RDW (OR 0.789, 95% CI 0.688-0.905, p=0.001), and sorafenib-mediated diarrhoea (OR 3.682, 95% CI 1.598-9.335, p=0.003, Table 3), but not NLR.

**Table 3:** Clinical variables significantly associated with 3 month and 12 month survival in patients on sorafenib therapy (n=232)

Clinical Variable	OR	95% CI	P-value	AUC	SensitivitySpecificityPPVNPP
3 month survival					
RD-CLIP≥70	0.017	0.004-0.075	<0.0001	0.808	Sensitivity 97.9%
Diarrhoea on sorafenib	4.990	1.774-14.04	0.002		Specificity 40.5%PPV 86.4%NPV 83.3%Correct identification 86.1%
12 month survival					
RD-CLIP ≥35	0.093	0.021-0.415	0.002	0.787	Sensitivity 44.4%
Recurrent HCC	3.943	1.652-9.408	0.002		Specificity 85.5%
Diarrhoea on sorafenib	1.777	0.810-3.899	0.152		PPV 55.6%NPV 79.0%Correct identification 73.6%

RD, red cell distribution width; CLIP, Cancer of the Liver Italian Program; HCC, hepatocellular carcinoma.

**Figure 1:**
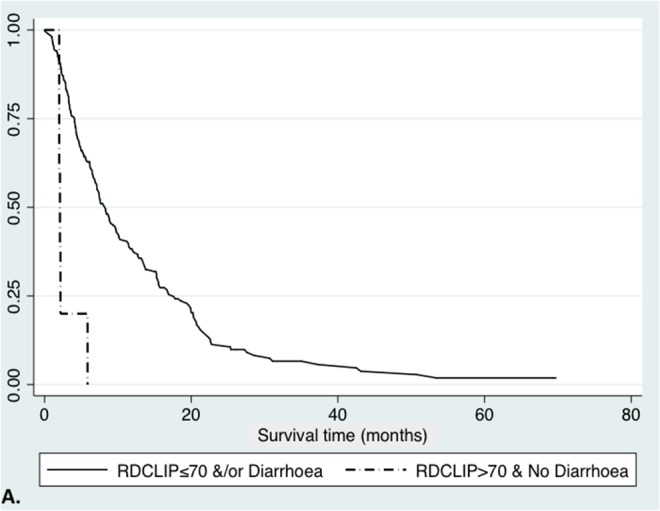

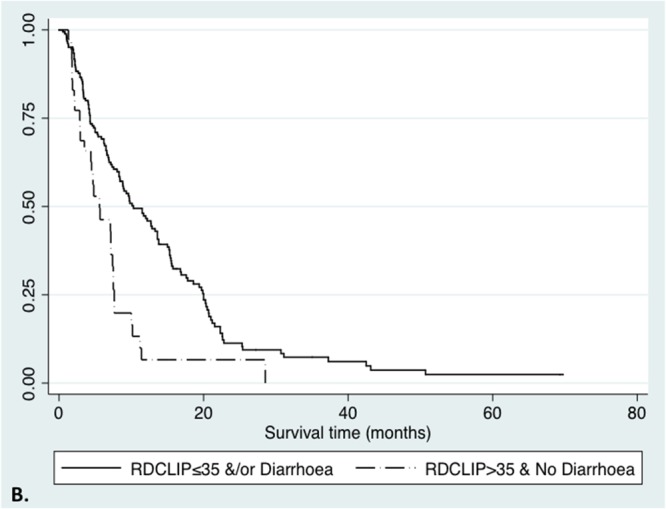
Survival curves in HCC patients receiving sorafenib, defined by RD-CLIP score and treatment-related diarrhoea **A**. Kaplan-Meier curve comparing RD-CLIP score greater than 70 and those without treatment-related diarrhoea, to those with RD-CLIP score less than or equal to 70 and/ or treatment-related diarrhoea (2.1 months (IQR 2.0-2.2 months) versus 8.3 months (IQR 4.1-17.6 months); Logrank p<0.0001, Wilcoxon p<0.0001). **B**. Kaplan-Meier curve comparing RD-CLIP score greater than 35 and those without treatment-related diarrhoea, to those with RD-CLIP score less than or equal to 35 and/ or treatment-related diarrhoea (5.6 months (IQR 2.8-18.6 months) versus 10.3 months (IQR 4.3-20 months); Logrank p<0.0001, Wilcoxon p=0.0003).

A combined logistic regression model of these three variables confirmed good predictive accuracy for 3-month survival, with an AUROC of 0.815 (95% CI 0.727-0.896). We then developed a novel prognostic index of baseline RDW multiplied by CLIP score (RD-CLIP), combined with sorafenib-related diarrhoea in a logistic regression model. Using variable cut-offs chosen to maximise sensitivity and specificity, a model using a baseline RD-CLIP score greater than or equal to 70and the presence of sorafenib-related diarrhoea had an AUROC for predicting three-month survival of 0.808 (95% CI 0.734-0.882), with sensitivity of 97.9%, specificity of 40.5%, positive predictive value of 86.4% and negative predictive value of 83.3%. This was superior to CLIP score alone (AUC=0.642) or BCLC score alone (AUC=0.579). Median survival time was 2.1 months (IQR 2-2.2 months) in patients with RD-CLIP score greater than or equal to 70 and no sorafenib-mediated diarrhoea, compared with 8.4 months (IQR 4.23-16.8 months) in patients who did not fulfil these criteria (Figure 1A).

Twelve-month survival was also independently associated with lower baseline RDW (OR 0.799, 95% CI 0.665-0.959, p=0.016), lower CLIP score (OR 0.663, 95% CI 0.469-0.937, p=0.020), and treatment-naïve compared with previously-treated HCC (OR 3.517, 95% CI 1.626-7.610, p=0.001) on logistic regression. A non-significant trend was observed between sorafenib-related diarrhoea and twelve-month survival (p=0.059). A predictive logistic regression model was optimised for sensitivity and specificity using a modified RD-CLIP cut-off of 35, recurrent HCC and no diarrhoea, with an AUROC of 0.787 (95% CI 0.718-0.857), suggesting moderate utility as a predictive model of twelve-month survival. Sensitivity for twelve-month survival was 44.4%, specificity 85.5%, positive predictive value (PPV) 55.6% and negative predictive value (NPV) was 79.0%. Again, this combination had higher accuracy for 12-month survival than either CLIP score (AUC=0.669) or BCLC score alone. (AUC=0.579).

### The association between sorafenib-mediated diarrhoea and RDW and survival is independent of dose reductions during treatment

Finally, an important consideration was whether the requirement for dose reductions during treatment was a confounding factor for the apparent association between sorafenib-mediated diarrhoea, RDW and survival. Median duration of sorafenib was 3.97 months (IQR 1.63-10.30 months). 261 (59.2%) patients commenced on 400mg sorafenib and 180 (40.8%) were commenced on 800mg daily, with those starting on lower dose rapidly titrated up to target dose. Data describing dose reductions during therapy were only available for 109 patients (25%). However, in a post-hoc analysis, sorafenib dose reduction was not associated with survival (Log-rank p=0.211; Wilcoxon rank-sum p=0.853) or with development of diarrhoea (χ^2^ p=0.122) or RDW (Wilcoxon rank-sum p=0.842). Though limited by small sample size and available data representing only 25% of the cohort, these data suggest sorafenib dose reduction was not a significant confounding factor for the association between diarrhoea or RDW and survival in patients with HCC treated with sorafenib in this study.

## DISCUSSION

Survival in advanced HCC is dependent upon the complex interplay between tumour burden and underlying liver disease, which ultimately determines patient outcome. Given the modest survival benefit offered by sorafenib and the frequent need for discontinuation due to poor tolerability, deterioration in liver function or progressive disease, prognostic markers to guide clinical decision-making in patients with advanced HCC are urgently sought. This large, prospective, multicentre study enrolling patients from a wide range of racial backgrounds and disease aetiologies shows for the first time that the combination of RDW and CLIP score together with treatment-related diarrhoea are superior markers for predicting overall, three-month and twelve-month survival compared with CLIP or BCLC score alone in patients with advanced disease on sorafenib.

RDW reflects inflammation-induced erythroid maturation impairment and has clinical utility as a marker of inflammation in both liver disease [15, 16, 17] and cancer [18]. Inflammation is central to liver damage, fibrogenesis and oncogenesis [10, 19], and inflammatory markers have prognostic value in HCC [20, 21]. Several indices, the Inflammation-based index, IBI; prognostic nutritional index, PNI; and modified Glasgow score, have prognostic utility for survival in HCC. However, no inflammatory scores have been systematically assessed in patients with advanced disease on sorafenib. Importantly, RDW has previously been validated as a marker of overall survival in HCC patients [15], lending credence to the association between RDW and survival in advanced HCC patients receiving sorafenib. This suggests inflammation is a key determinant of survival in advanced HCC, which fits with the known driving role of inflammation in hepatocarcinogenesis [10, 19]. RDW also reflects nutritional status, itself a marker of performance status in cancer and also systemic immune function [15]. Moreover, RDW is an inexpensive and accessible biomarker in patients with HCC receiving sorafenib, as it is already provided in the full blood count.

A key finding of this study is that overall survival, 3 month and 12 month survival were associated with diarrhoea as a side effect of sorafenib use, but not skin toxicities as previously reported. A number of studies have reported off-target effects of sorafenib as prognostic factors, including diarrhoea and skin toxicities [22–31]. Bettinger et al [29] al reported survival was twice as long in patients who developed diarrhoea on sorafenib compared to those who did not (7.1 months versus 14.1 months), findings that have been replicated by others [26, 32]. The current study is the largest to date reporting the association between treatment-related diarrhoea and survival, lending strength to these findings.

It is likely that diarrhoea reflects systemic drug levels and drug activity [23, 25, 26, 31, 32]. Evidence suggests that there is survival benefit of sorafenib even in patients who cease drug due to toxicities, compared with those who cease sorafenib due to progressive disease [25], findings that were echoed in our study. Another potential mechanism of improved survival may be through the effects of diarrhoea on the gut microbiome and bacterial translocation, both key determinants of survival in cirrhosis with portal hypertension [33, 34]. Sorafenib-induced diarrhoea may lead to alterations in gut flora or reduction in the load of nitrogenous commensal bacteria, therefore reducing bacterial translocation and ammonia absorption akin to the therapeutic effects of lactulose or rifaximin on encephalopathy [35].

Though 50% of our patients reported hand-foot skin toxicity, there was no association with survival. However, skin rash other than hand-foot syndrome was uncommon in the cohort (13%) and grade 3-4 HFS was rare, and may explain the lack of association found between skin toxicity and survival in this study. Hypertension has been reported as a prognostic variable for survival [26], however we did not replicate this finding, in line with another previous negative report [23].

CLIP score, but not BCLC score, was prognostic for survival on multivariate analysis. There is good evidence for the prognostic potential of both CLIP and BCLC scores in HCC [1, 36] and they have traditionally been used to stratify those who are most likely to benefit from sorafenib [1, 36]. Key features of both scores, including tumour morphology, size, AFP levels, metastases and PVT have all been shown to predict survival in patients on sorafenib [3, 4, 25, 27, 33, 37–39]. The main differences between BCLC and CLIP scores are greater weight given to CTP class and inclusion of AFP in CLIP [1]. The discrepancy between our finding regarding BCLC score and other reports [39, 40] is not surprising given the relative homogeneity of our patient cohort, with inherent over-representation of patients with well-compensated liver disease and advanced HCC. These data also suggest that AFP is an important prognostic factor for survival in patients on sorafenib, particularly in patients with advanced disease, which is supported by data from the study of the VEGF2 inhibitor, ramucirumab, where patients with higher AFP levels had shorter overall survival [41]. Whilst BCLC remains undoubtedly a useful validated system to prioritize treatment allocation, our study suggests a better stratifying role for CLIP in the prognostic assessment of patients with advanced HCC receiving sorafenib, in line with previous evidence gathered in patients with advanced disease.

Current prognostic scores such as BCLC and CLIP do not include markers of inflammation [1]. However, by combining red cell distribution width with CLIP score and sorafenib-mediated diarrhoea, 3-month survival was predicted with good accuracy (AUC 0.808) and 12 month survival with moderately good accuracy (AUC 0.787), with greater accuracy than CLIP score or BCLC scores alone. Importantly, baseline RD-CLIP and diarrhoea had very high sensitivity for predicting 3-month survival, with a relatively high PPV and NPV in this study population. Our data suggest the addition of inflammatory parameters to prognostic assessments such as CLIP or BCLC significantly improves prediction of patients most likely to benefit from sorafenib, which will inform discussions with patients experiencing side effects and considering treatment cessation or changing to second-line agents.

There are several limitations to our study. This is a pilot cohort study powered to detect clinical variables associated with overall survival that may prove useful biomarkers of sorafenib survival in future studies. These findings therefore require validation in independent cohorts before they can be incorporated into current HCC management guidelines, however the data are highly suggestive of the merit of further validation studies. We did not evaluate dynamic changes in clinical parameters over time to confirm ongoing prediction of survival and response to treatment, which would be useful to confirm their utility throughout duration of sorafenib therapy. Moreover, diarrhoea is a more subjective measure than RDW or CLIP score for the purposes of survival prediction. However, the current study includes a large cohort of patients and is well-powered to detect significant associations between clinical variables and overall survival, which lends considerable weight to our findings.

## MATERIALS AND METHODS

This is a prospective, observational cohort study of prognostic factors in patients receiving sorafenib for advanced HCC. The primary study endpoint was overall survival (OS) after commencing sorafenib, censored at either death or end date of study follow up (30 March 2015). Clinical factors related to OS and disease progression were determined.

Patients with HCC were consecutively recruited to the study from three tertiary centres with specialist multidisciplinary services for HCC management: Osaka, Japan (183 patients, 41.4%); Novara, Italy (156, 35.4%) and Hammersmith Hospital, Imperial College, London UK (103, 23.3%) from January 1, 2008 until censorship for death or loss to follow up, or the study end date of December 31, 2015. Demographic and clinical data, blood tests and imaging results were collected prospectively. All patients had a diagnosis of HCC based either on imaging or histologic criteria according to international guidelines [1]. Patients were staged using the Barcelona Clinic Liver Cancer (BCLC) and CLIP scores, both of which describe liver functional impairment using the Child Turcotte Pugh score (CTP) [1].

The study was approved by local institutional ethics committees and conducted in accordance with the Declaration of Helsinki (update 2004).

### Sorafenib treatment

Patients were commenced on sorafenib therapy in accordance with BCLC guidelines (BCLC stage C, or BCLC stage B if other factors precluded use of loco-regional therapies). All patients in the study were unsuitable for surgical resection of HCC. Patients with decompensated liver disease and performance scores ≥2 were excluded. Duration, dose modifications and tolerability to sorafenib were recorded. Cause for cessation of therapy (toxicity, patient preference, disease progression or death) was also recorded. Disease progression was defined by mRECIST criteria [42].

### Statistical methods

Variables were described using mean and standard deviation or median and interquartile range (IQR), with natural logarithmic transformation of skewed data. NLR was calculated by dividing the neutrophil count by the lymphocyte count. Univariate analysis of variables associated with survival was performed using Log-rank testing. Multivariate analysis was performed using Cox proportional hazards regression modelling using backward elimination and likelihood ratio testing, including variables significantly associated with survival on univariate analysis. Proportional hazards was confirmed using Schoenfeld residuals and visually using log-log plots. Prognostic model building utilised logistic regression modelling with stepwise backward elimination and likelihood ratio testing. Receiver Operator Curve (ROC) analysis determined diagnostic accuracy of regression models for predicting three-month survival and twelve-month survival. All analyses were performed using STATA version 12.1 (Stata Corporation, College Station, Texas, USA).

## CONCLUSION

A novel prognostic index of CLIP score combined with RDW and the presence of treatment-related diarrhoea had good accuracy for determining overall, 3 month and 12 month survival in patients who have commenced sorafenib. Patients with RD-CLIP scores ≥ 70 and no treatment-related diarrhoea had median survival of 2.1 months compared with 8.4 months in those who did not fulfil these criteria. The addition of simple, easy-to-measure RDW and sorafenib-related diarrhoea to current prognostic algorithms in advanced HCC management improves accuracy for predicting survival benefit of sorafenib in patients with advanced HCC and warrants further validation in future studies.

## SUPPLEMENTARY MATERIALS FIGURES AND TABLES




