# Supplementary material for: Integration of the cancer-related inflammatory response as a stratifying biomarker of survival in hepatocellular carcinoma treated with sorafenib
Source: Oncotarget. 2017 Feb 14;8(22):36161–70. doi: 10.18632/oncotarget.15322 (PMC5482646; doi:10.18632/oncotarget.15322)
Supplement: Supplementary file 1 [file oncotarget-08-36161-s001.pdf]

Integration of the cancer-related inflammatory response as a stratifying biomarker of survival in hepatocellular carcinoma treated with sorafenib

SUPPLEMENTARY METHODS

All three centres had similar patient cohorts and utilized similar treatment criteria and collected data using the same methodology.

Diagnosis of sorafenib-mediated toxicities

All toxicities developing on sorafenib treatment were attributed to sorafenib if they were temporally associated with commencement or dose increase in sorafenib and occurred in the absence of obvious concomitant disease (as evidenced on clinical examination and blood or body fluid tests). Diarrhoea was identified as sorafenib-related when occurring in association with starting or increasing sorafenib dose, in the absence of a new increase in lactulose dosing and infective features (clinically and biochemical evidence of inflammation and/or a faecal specimen positive for viral or bacterial culture).

Supplementary Table 1: Univariate analysis of clinical variables associated with overall survival in patients on sorafenib therapy (n=442)

See Supplementary File 1
