# Supplementary material for: Integration of the cancer-related inflammatory response as a stratifying biomarker of survival in hepatocellular carcinoma treated with sorafenib
Source: Oncotarget. 2017 Feb 14;8(22):36161–70. doi: 10.18632/oncotarget.15322 (PMC5482646; doi:10.18632/oncotarget.15322)
Supplement: Supplementary file 2 [file oncotarget-08-36161-s002.docx]

**Supplementary Table 1. Univariate analysis of clinical variables associated with overall survival in patients on sorafenib therapy (n=442)**

| **Clinical Variable** | **OR** | **95% CI** | **P-value** |
| --- | --- | --- | --- |
| Female gender (n=442) | 0.917 | 0.696-1.209 | 0.540 |
| Age (n=442) | 0.986 | 0.974-0.997 | **0.013** |
| ALT (μmol/L) (n=438) | 1.003 | 1.002-1.004 | **<0.0001** |
| Total bilirubin (μmol/L)(n=442) | 1.003 | 1.002-1.005 | **0.003** |
| Albumin (g/L) (n=441) | 0.974 | 0.957-0.991 | **0.002** |
| CTP score (n=441) | 1.242 | 1.120-1.379 | **<0.0001** |
| Neutrophils (n=438) | 1.181 | 1.124-1.240 | **<0.0001** |
| Lymphocytes (n=438) | 0.786 | 0.654-0.946 | **0.011** |
| Platelets (n=440) | 1.002 | 1.001-1.003 | **0.001** |
| NLR (n=438) | 1.244 | 1.182-1.309 | **<0.0001** |
| Red cell distribution width (n=425) | 1.110 | 1.070-1.151 | **<0.0001** |
| Viral hepatitis (n=213) | 1.342 | 0.987-1.825 | **0.061** |
| Alcohol Liver Disease (n=210) | 0.844 | 0.615-1.157 | 0.292 |
| Tumour morphology (n=438)  <50% uninodular  <50% multinodular  >50% multinodulat | 1.00  1.019  1.975 | 0.735-1.414  1.375-2.836 | 0.909  **<0.0001** |
| AFP (ng/mL) (n=420) | 1.000005 | 1.000004-1.000006 | **<0.0001** |
| Portal vein thrombosis (n=442) | 1.735 | 1.364-2.207 | **<0.0001** |
| Tumour size (cm) (n=427) | 1.106 | 1.073-1.140 | **<0.0001** |
| Number of nodules (n=380)  1-3 nodules  4-6 nodules  7-10 nodules  Multinodular  Diffuse | 1.000  0.341  0.361  0.507  0.468 | 0.108-1.079  0.113-1.153  0.159-1.612  0.117-1.874  0.313-5.042 | 0.067  0.085  0.250  0.283  0.748 |
| Metastases (n=442) | 1.493 | 1.164-1.914 | **0.002** |
| BCLC score (n=428)  1  2  3  4  5  6  7 | 1.000  1.610  3.403  0.561  1.407  1.602  4.698 | 0.205-12.623  0.740-15.646  0.254-1.237  0.735-2.694  0.843-3.044  1.460-15.123 | 0.651  0.116  0.152  0.392  0.150  **0.009** |
| CLIP score (n=276)  0  1  2  3  4  5  6 | 1.00  1.141  1.004  1.900  2.352  5.219  33.030 | 0.620-2.097  0.550-1.833  1.021-3.538  1.177-4.701  2.313-11.779  4.015-271.685 | 0.671  0.989  **0.043**  **0.015**  **<0.0001**  **0.001** |
| Primary versus recurrent disease (n=204) | 0.630 | 0.453-0.875 | **0.006** |
| Sorafenib cessation due to side effects (n=438) | 0.839 | 0.642-1.098 | 0.201 |
| Hand-Foot syndrome (n=287) | 0.431 | 0.326-0.572 | **<0.0001** |
| Diarrhoea (n=261) | 0.546 | 0.408-0.730 | **<0.0001** |
| Anorexia (n=205) | 0.806 | 0.589-1.103 | 0.178 |
| Hypertension (n=168) | 0.313 | 0.205-0.478 | **<0.0001** |
| Mucositis (n=118) | 0.600 | 0.294-1.222 | 0.159 |

IQR, interquartile range; ALT, alanine aminotransgerase; NLR, Neutrophil to Lymphocyte ratio; AFP, alpha-fetoprotein; BCLC, Barcelona Clinic Liver Cancer score; CLIP, Cancer of the Liver Italian Program score.
